# Genetic Evaluation of Barrel Racing Performance in Quarter Horses

**DOI:** 10.1111/jbg.12934

**Published:** 2025-03-01

**Authors:** Mário Luiz Santana, Thiago Garcia Botelho Franco, Annaiza Braga Bignardi

**Affiliations:** ^1^ Grupo de Melhoramento Animal de Mato Grosso (GMAT), Instituto de Ciências Agrárias e Tecnológicas Universidade Federal de Rondonópolis (UFR) Rondonópolis Brazil; ^2^ EquiVet Medicina Equina Rondonópolis Brazil

**Keywords:** equestrian sport, genetic parameters, heritability, random regression, speed

## Abstract

Barrel racing is a competitive timed rodeo event that challenges horses and riders to complete a cloverleaf pattern around three barrels in the fastest time possible. In this study, we aimed to estimate the genetic parameters of barrel racing time (BRT) and evaluate the most suitable statistical model for its analysis. We compared a repeatability model and three random regression models (RRM) to analyse the longitudinal BRT data in Brazilian Quarter Horses. A total of 356,877 BRT records from 14,108 horses that competed in various events held across Brazil between 2010 and 2024 were analysed. The cubic RRM provided the best fit to the data, and therefore, the results from this model were presented in detail. Heritability estimates for BRT varied by age (0.15–0.24), with the highest estimates observed between 36 and 54 months, suggesting that selection at younger ages could be most effective. Genetic correlations between BRT at different ages were generally strong (> 0.8). The lowest mean genetic correlation of 0.65 (0.09) was observed between BRT at 36 and 144 months of age. Thus, selecting the best‐performing horses at younger ages should result in favourable genetic progress at older ages. Phenotypic trends showed an improvement in BRT over the years, although no significant genetic progress was observed, likely due to the absence of an official breeding programme and the lack of use of estimated breeding values for BRT. These findings highlight the need for a more strategic approach to genetic selection in Quarter Horses to optimise BRT performance. The substantial genetic variation identified for BRT indicates that, if properly exploited, this trait could be significantly improved in the future, ultimately enhancing competition outcomes for Brazilian Quarter Horses in barrel racing.

## 1 Introduction

Barrel racing is a timed rodeo event in which horses and riders navigate a cloverleaf pattern around three barrels as quickly as possible. Competitors start the course by selecting either the right or left barrel to circle first, then proceed to the opposite barrel, and finally complete the circuit by rounding the third barrel before sprinting through the center to stop the timer. Precision and speed are essential, as knocking over a barrel incurs a 5 s penalty. Originally developed as a women's rodeo event in the United States during the 1940s (Stricklin 1997), barrel racing has since evolved to include participants of all genders, ages and skill levels (Holbrook and Schoonover 2024).

In Brazil, barrel racing was introduced in the early 1970s with the arrival of the American Quarter Horse breed. Known for their agility, speed, and quick turns, Quarter Horses are particularly suited for barrel racing. Today, barrel racing has gained widespread popularity, with well‐organised events and substantial prize money offered in several countries (Dabareiner et al. 2005; Holbrook and Schoonover 2024). By 2024, over 6.2 million dollars in prize money had been distributed across events in Brazil (SGP 2024). This highlights the growing economic and cultural significance of barrel racing within the equine industry.

The economic impact of barrel racing extends beyond prize money, as it drives investments in breeding, training, and equine‐related industries. Estimating genetic parameters for performance traits in Quarter Horses could provide valuable insights for breeders and stakeholders, enabling the selection of superior animals and optimising breeding strategies. This, in turn, could enhance the competitiveness of the sport and increase the economic returns for participants and the industry.

Despite the importance of barrel racing, no study to date has estimated genetic parameters for Quarter Horse performance in these competitions. Identifying traits with the potential to respond to selection is critical for the development of genetic improvement programmes in horses (Velie et al. 2015). Racing performance in horses has been reported as heritable (Bakhtiari and Kashan 2009; Faria et al. 2023; Sharman and Wilson 2023), and the longitudinal nature of several traits of interest for selection allows for the application of random regression models (Gómez et al. 2010). According to Bugislaus et al. (2006) and Buxadera and da Mota (2008), random regression models are highly recommended for evaluating racehorse performance and enable improved selection accuracy over age.

The primary objective of the present study was to estimate, for the first time, genetic parameters for barrel racing performance in Brazilian Quarter Horses using a random regression model. We compared different random regression models depending on the age of the animals and a repeatability model to provide support for an official genetic evaluation. Additionally, we investigated the trends of barrel racing performance over the years to identify any evidence of selection practices within the population.

## 2 Materials and Methods

### 2.1 Data and Quality Control

**FIGURE 1:**
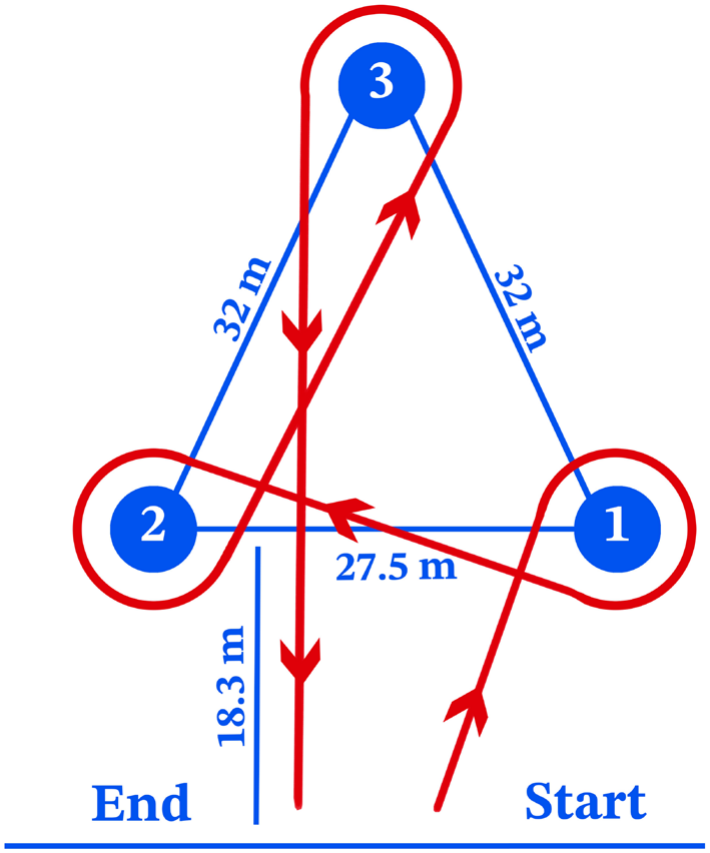
An illustrative diagram of the course to be followed (red line and arrows) individually by Quarter Horses in barrel racing competitions. The horizontal blue line represents the start and finish of the course, and each number corresponds to one of the three barrels. [Colour figure can be viewed at wileyonlinelibrary.com]

**FIGURE 2:**
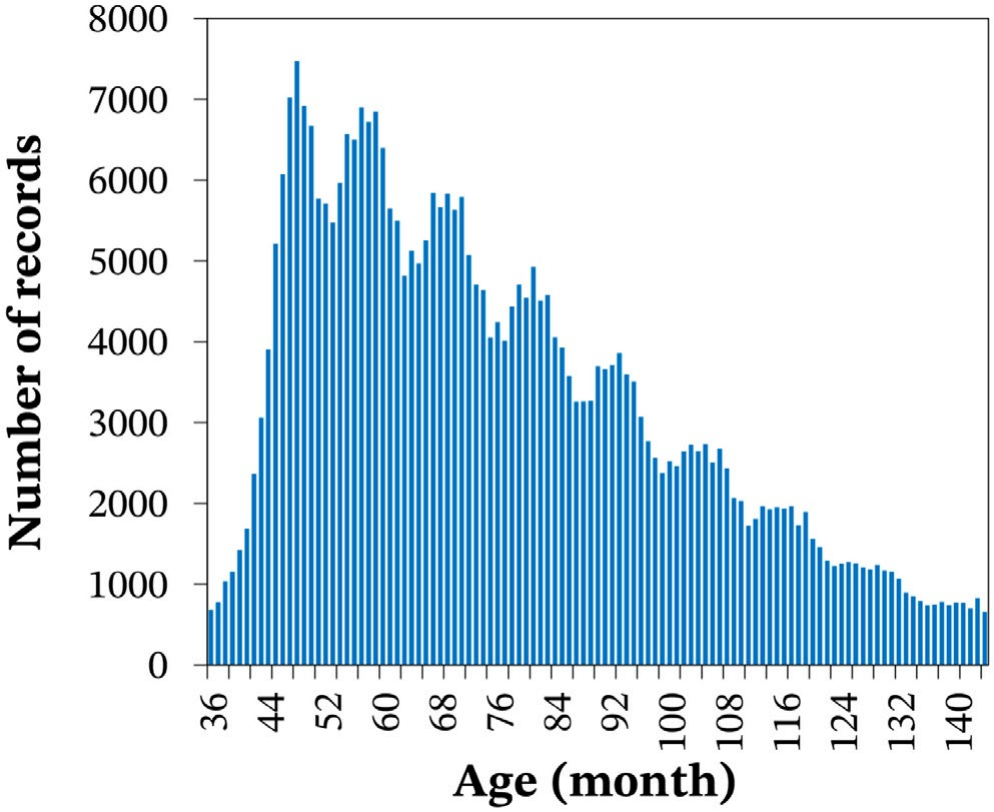
Distribution of barrel racing time records for Quarter Horses according to the age of the animals. [Colour figure can be viewed at wileyonlinelibrary.com]

The racing performance records of 14,108 Quarter Horses were provided by the *Sistema SGP de Gerenciamento de Provas* (https://www.sgpsistema.com) and included data from 13,035 races held between 2010 and 2024 across 23 states in all regions of Brazil. The five states with the highest number of horse performance records were São Paulo (61.1%), Paraná (13.7%), Rio de Janeiro (7.6%), Minas Gerais (5.6%) and Mato Grosso (2.6%). The phenotype analysed in this study was barrel racing time (BRT), measured in seconds. The course covered by the horses for BRT measurement is illustrated in Figure 1. According to barrel racing regulations, horses and riders are penalised by 5 s for each barrel knocked down; however, information on penalties was not available in our dataset. Therefore, based on a prior data analysis, we decided to exclude records of horses that were potentially penalised. We considered valid BRT records only those with times up to 4.99 s above the record time of 16.109 s for the event in Brazil. This restriction led to the exclusion of approximately 15% of the originally available records. Additionally, records were excluded for races with fewer than 10 animals, horses with unknown sire or dam, records from horses younger than 36 months or older than 144 months, and riders who participated in fewer than three races. A detailed description of the data after quality control and the pedigree information of the horses is provided in Table 1. The distribution of records according to the age of the animals is shown in Figure 2.

**TABLE 1:** Summary of data structure for barrel racing time of Quarter Horses.

Statistics	Values
Horses in the pedigree file, *n*	31,902
Records, *n*	356,877
Horses with records, *n*	14,108
Mean number of records per horse, *n*	25.29
Horses with up to 10 records, %	42.42
Horses with 11 to 20 records, %	20.71
Horses with 21 to 30 records, %	11.31
Horses with 31 to 40 records, %	7.07
Horses with 41 to 50 records, %	4.66
Horses with more than 50 records, %	13.83
Male records, *n*	175,093
Female records, *n*	181,784
Stallions with progeny record, *n*	2122
Stallions with own record, *n*	441
Mares with progeny record, *n*	7268
Mares with own record, *n*	1455
Races, *n*	13,035
Riders, *n*	5324
Mean and standard deviation of the barrel racing time, *s*	17.985; 0.749
Minimum and maximum of the barrel racing time, *s*	16.109; 21.099

### 2.2 Analysis and Models

An initial data analysis was conducted to identify the key factors influencing horse performance. A simple linear model was applied, which included the fixed effects of the contemporary group, animal age at the competition date (in months), sex and the random effects of the animal and residual. Barrel racing competitions encompass categories and subcategories generally defined based on the horse's age and the rider's age and gender. In the present study, all categories and subcategories from events held on courses with official measurements were included. Contemporary groups were defined by combining information from each event, including location, category, subcategory and competition date. As such, the contemporary group was designed to account for effects such as competition level, track conditions, and the thermal environment on the date of each event. All fixed effects mentioned above were significant (*p* < 0.001) and retained for subsequent analyses. To examine the phenotypic performance response patterns according to the sex and age of the horses, we calculated the least squares means.

The (co)variance components and genetic parameters were estimated using a repeatability animal model (M0) and three random regression animal models (RRM) of different orders: linear (M1), quadratic (M2) and cubic (M3). The evaluated models included the same effects and differed only in the modelling of the additive genetic effect and the permanent environmental effect of the animal. The RRM can be described as follows:

$$y_{\mathrm{ikl}}=\sum_{m=0}^{3}\beta_{m}\emptyset_{m}\left(t\right)+\sum_{m=0}^{n}\alpha_{\mathrm{im}}\emptyset_{m}\left(t\right)+\sum_{m=0}^{n}\gamma_{\mathrm{jm}}\emptyset_{m}\left(t\right)+b_{k}+f_{l}+e_{\mathrm{ikl}}$$

where, $y_{\mathrm{ijk}}$ was the phenotype of the *i*‐th horse; $\beta_{m}$ is the *m*‐th “fixed” regression coefficient to describe the population performance according to the age *t* (36 to 144 months) $;\emptyset_{m}\left(t\right)$ was the *m*‐th Legendre orthogonal polynomial (*n* = 1, 2 or 3) corresponding to the age *t*; $\alpha_{\mathrm{im}}$ and $\gamma_{\mathrm{jm}}$ were the random regression coefficients for the additive genetic and animal permanent environmental effects, respectively; $b_{k}$ was the uncorrelated random effect of the rider; $f_{l}$ was the set of systematic effects (contemporary group and sex) as described above, and $e_{\mathrm{ikl}}$ was the residual effect. Information regarding the training regimen was not available. Nevertheless, given that this effect may influence multiple records from the same animal, it is anticipated that at least a portion of its impact is captured by the random regression function for the permanent environmental effect. Further details on the application of RRM in animal breeding can be found in Oliveira et al. (2019). A heterogeneous residual variance structure (four age classes) was adopted for both repeatability and RRM: 1: ≥ 36 to < 42; 2: ≥ 42 to < 54; 3: ≥ 54 to < 72; and 4: ≥ 72 months. These four classes were defined in a previous analysis using a repeatability model and 19 six‐month age classes. Based on the similarity of the residual variance estimates obtained, the four classes were defined appropriately.

The analyses were performed based on a Bayesian approach. Chains consisting of 300,000 samples, a burn‐in period of 100,000 samples, and a thinning interval of 20 were used. Convergence was visually assessed using trace plots to evaluate the mixing of the chains. The remaining 10,000 samples were utilised to compute the genetic parameters. Data handling, calculation of least square means and regression analyses were conducted using the R programming language. The estimation of (co)variance components and genetic parameters was performed using the GIBBSF90+ program (Misztal et al. 2014).

### 2.3 Model Comparison

The deviance information criterion (DIC) was used to evaluate and compare model fit (Spiegelhalter et al. 2002). Lower DIC values indicate a better‐fitting model.

### 2.4 Genetic Trend

We assessed the posterior means of estimated breeding value (EBV) for each horse across all ages as:

$${\mathrm{EBV}}_{i}=t^{\prime }{\hat{u}}_{i},$$

where *t* was a vector with the elements equal to the sum of the orthogonal polynomial from age 36–144 months, and ${\hat{u}}_{i}$ was the vector of EBV of the regression coefficients for horse *i*. The mean EBV for all horses was calculated according to the birth year. A simple linear regression model was fitted to these means. Thus, the *p*‐value for slope term tests the null hypothesis that the coefficient is equal to zero (no trend).

## 3 Results

### 3.1 Effect of Age and Sex on Horse Performance

**FIGURE 3:**
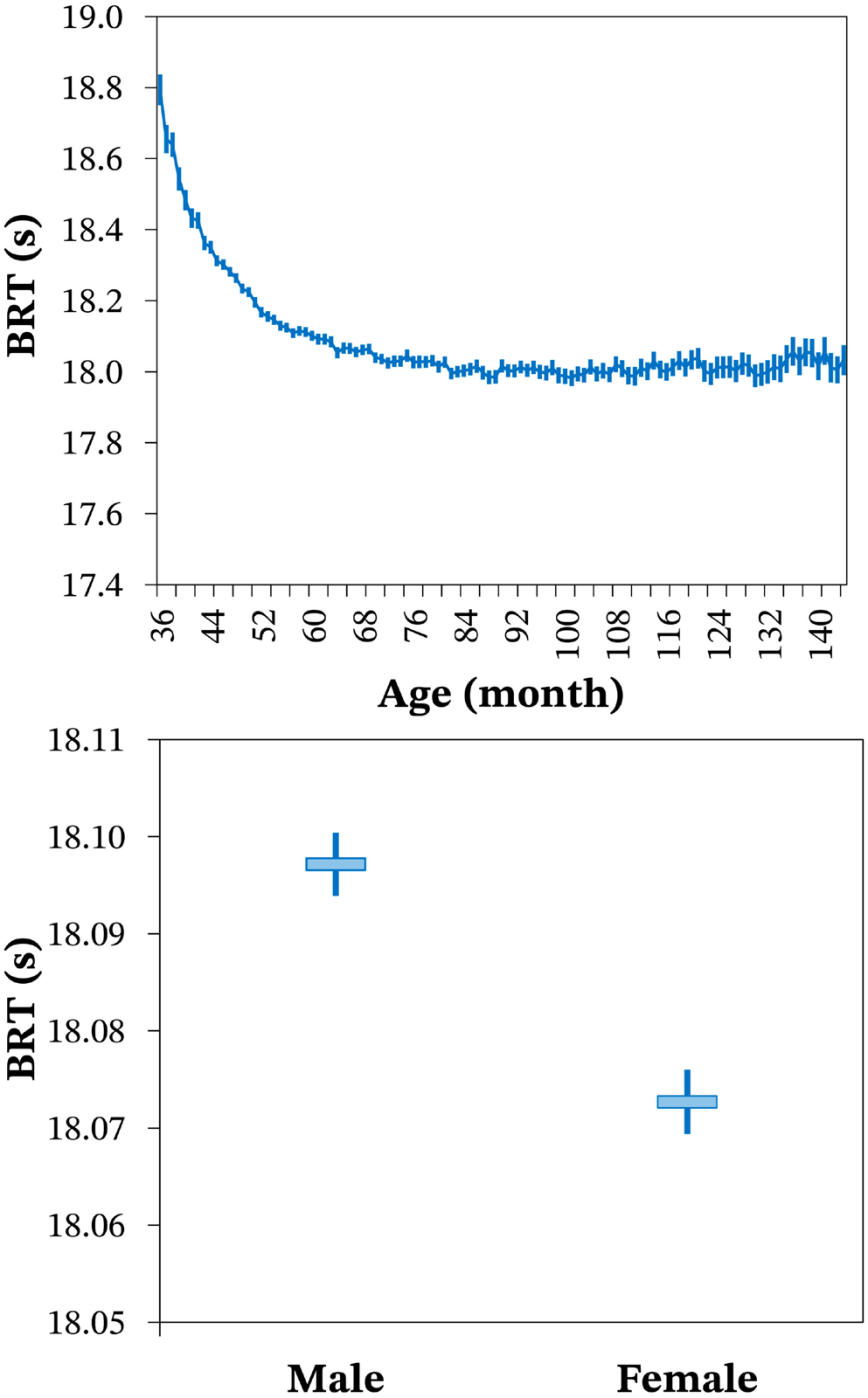
Least squares means (horizontal lines or bars) and 95% confidence interval (vertical bars) for barrel racing time (BRT) according to age (above) or sex (below) of Quarter Horses. [Colour figure can be viewed at wileyonlinelibrary.com]

The average age of horses participating in barrel racing events was approximately 76.5 months, equivalent to 6.37 years. The most common age (mode) of horses in competitions was 48 months. Horse age significantly influenced (*p* < 0.001) BRT, with younger animals exhibiting poorer performance (higher times). As horse age increased, a progressive improvement in average performance was observed up to approximately 73 months. This minimum value was determined through a cubic function fitted to the least squares means, after which performance stabilised (Figure 3). Sex also significantly influenced (*p* < 0.001) BRT, with females exhibiting an average performance that was 0.025 s better than males (Figure 3).

### 3.2 Model Comparison

The DIC values obtained as deviations from the best‐fitting model were 28,907, 10,643, 4773, and 0 for M0, M1, M2 and M3, respectively. Therefore, the cubic RRM (M3) was identified as the most suitable model for analysing the BRT of Quarter Horses. Consequently, most of the results presented below are based solely on M3.

### 3.3 Genetic Parameters

**FIGURE 4:**
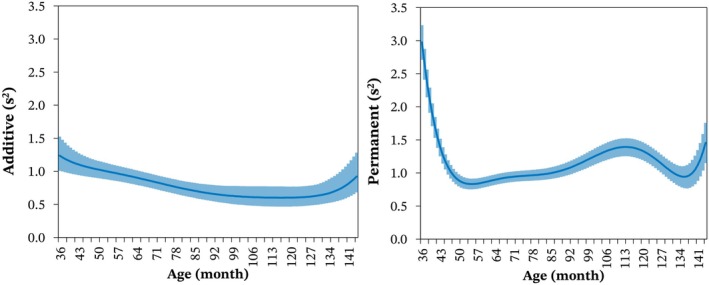
Posterior means (line) and 95% credibility intervals (shade) of estimates for additive genetic variance (left) and permanent environmental variance (right) for barrel racing time according to the age of Quarter Horses. [Colour figure can be viewed at wileyonlinelibrary.com]

In general, the additive genetic variance for BRT was higher at younger ages and tended to decrease at older ages (Figure 4). The variance of the permanent environmental effect of the animal decreased at younger ages until approximately 50 months, then slightly increased until around 115 months. For ages beyond 115 months, the variance of the permanent environmental effect of the animal decreased.

**FIGURE 5:**
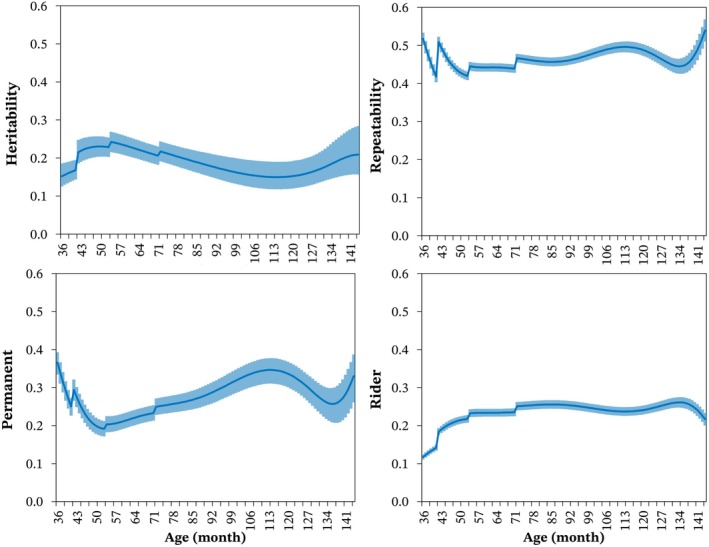
Posterior means (line) and 95% credibility intervals (shade) of estimates for heritability, repeatability, permanent environmental variance (expressed as a proportion of phenotypic variance), and rider variance (expressed as a proportion of phenotypic variance) for barrel racing time according to the age of Quarter Horses. [Colour figure can be viewed at wileyonlinelibrary.com]

As a reference, the heritability estimates for BRT obtained using M0 ranged from 0.14 (0.01) for ages up to 42 months to 0.23 (0.01) for ages over 72 months. In contrast, with M3 (Figure 5), the heritability estimate was 0.15 (0.01) at 36 months, reached a maximum of 0.24 (0.01) at 54 months, and decreased at older ages. A slight increase in heritability estimates was observed for ages beyond 115 months.

The estimates of repeatability and the permanent environmental effect of the animal, expressed as a proportion of the phenotypic variance, were higher at younger (36 months) and older (144 months) ages (Figure 5). A slight increase in these estimates was observed for ages between 50 and 115 months. The average repeatability estimate across the age trajectory was 0.46. The average estimate for the permanent environmental effect as a proportion of the phenotypic variance was 0.28. Similarly, the rider effect as a proportion of the phenotypic variance was 0.23, with lower values observed at very young or advanced ages. The posterior mean of residual variance was 2.98 (0.63) s^2^ for the age class below 42 months, which decreased to 1.07 (0.04) s^2^ for the age class of 72 months and older.

**FIGURE 6:**
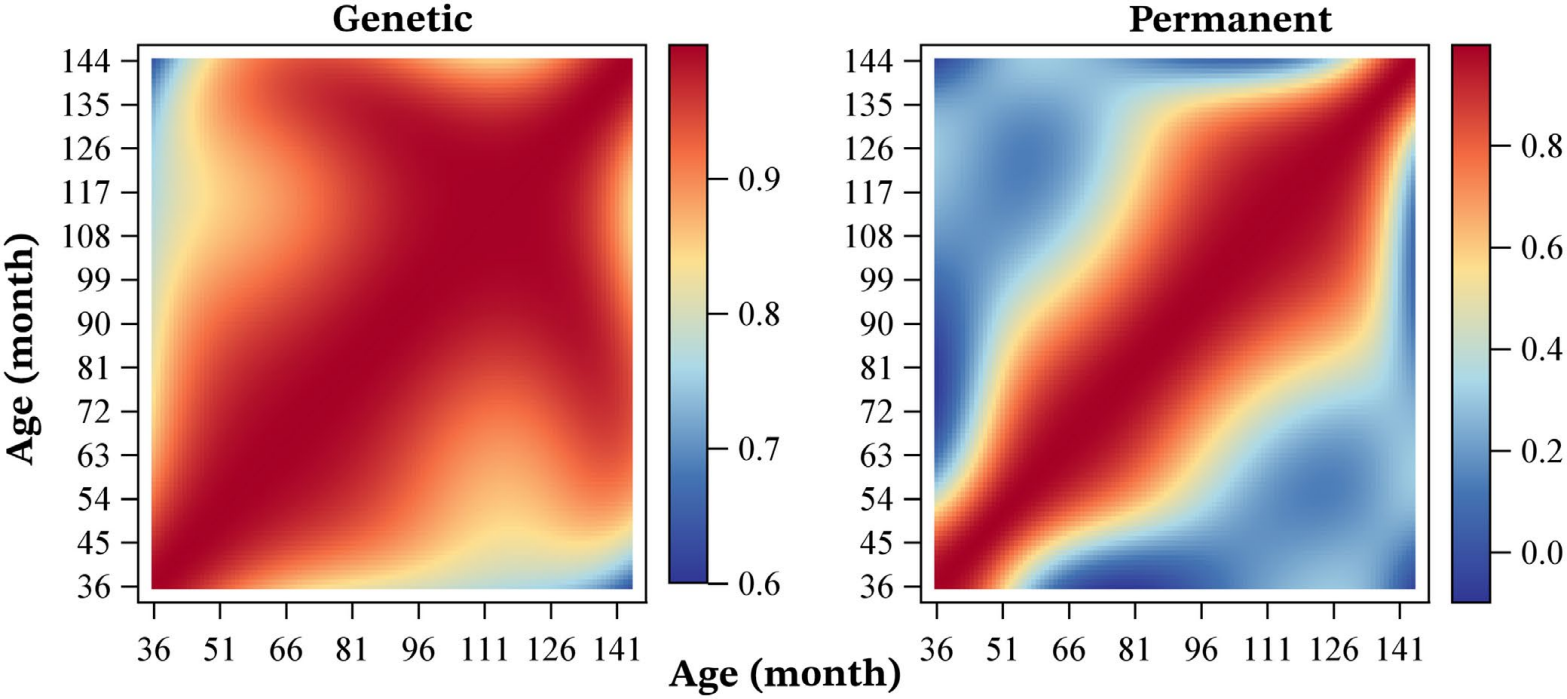
Posterior means of estimates for genetic correlation (left) and permanent environmental correlation (right) for barrel racing time according to the age of Quarter Horses. [Colour figure can be viewed at wileyonlinelibrary.com]

In general, the posterior mean of additive genetic correlations for BRT across different ages was strong (> 0.8), as shown in Figure 6. For instance, the mean genetic correlation estimates for BRT between 36 months and 48, 60 and 72 months were 0.96 (0.02), 0.89 (0.04) and 0.85 (0.05), respectively. The lowest mean genetic correlation of 0.65 (0.09) was observed between BRT at 36 and 144 months of age. In contrast, the posterior mean estimates of permanent environmental correlations for BRT across divergent ages were low to moderate (< 0.6), as illustrated in Figure 6. For example, the permanent environmental correlations between BRT at 36 months and 48, 60, 72 and 144 months were 0.76 (0.06), 0.20 (0.03), −0.05 (0.01) and − 0.03 (0.06), respectively.

### 3.4 Genetic Trend

**FIGURE 7:**
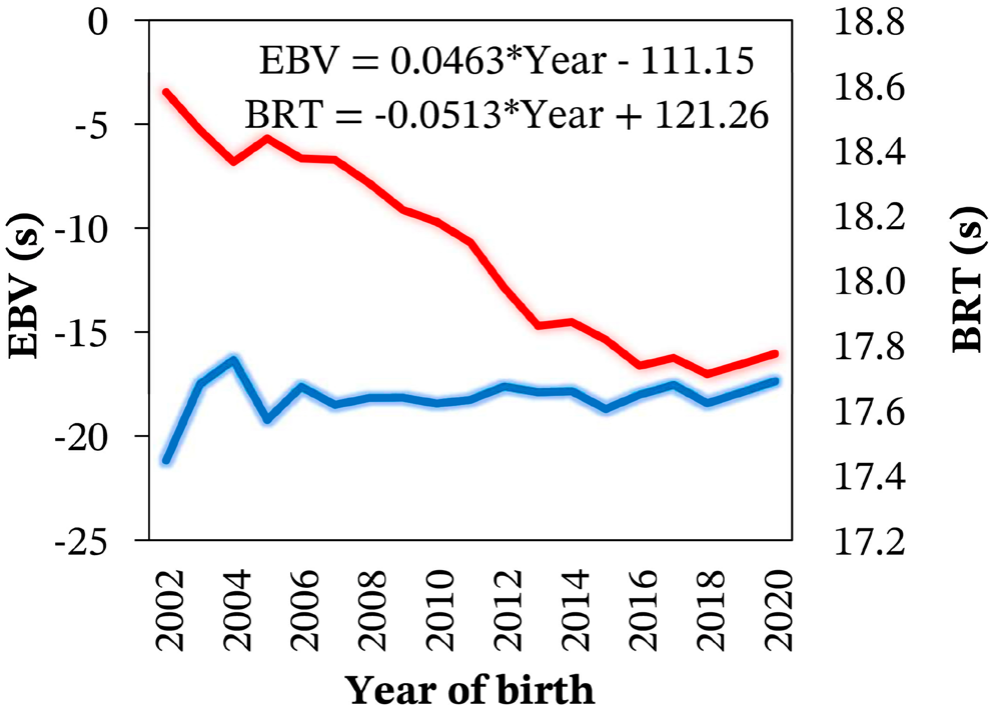
Means of estimated breeding values (EBV, left axis, blue line) and phenotypic performance (right axis, red line) for barrel racing time (BRT) according to the year of birth of Quarter Horses. Simple linear regression equations were presented for each trend. [Colour figure can be viewed at wileyonlinelibrary.com]

No significant (p > 0.05) genetic evolution for BRT was observed over the years analysed, as shown in Figure 7. In contrast, we observed an improvement (p < 0.001) in the average phenotypic performance for BRT of −0.0513 s/year between 2002 and 2020.

## 4 Discussion

Barrel racing involves riders and their horses navigating a cloverleaf pattern around three barrels, with the fastest time determining the winner. The sport has become an essential part of the rodeo culture, particularly for women, empowering them to excel in equestrian sports. The Quarter Horse has played a pivotal role in the development of barrel racing, becoming the primary breed used in the sport. Despite the longstanding and significant history of barrel racing, there is limited scientific knowledge regarding the genetic improvement of Quarter Horses specifically for this equestrian sport. Genetic parameter estimation studies in Quarter Horses have been conducted since the late 1980s (Buttram et al. 1988; Wilson et al. 1988). However, these studies predominantly focus on traits such as speed, ranking or earnings (da Gama et al. 2016; Faria et al. 2023). Barrel racing performance demands not only speed but also exceptional agility and precise movement control to complete the course in the shortest time without knocking over barrels. Thus, barrel racing performance is a complex trait influenced significantly by non‐genetic factors.

We identified a significant impact of age on the performance of horses in barrel racing, with younger horses generally exhibiting poorer performance. This finding aligns with Villela et al. (2002), Eki̇z et al. (2005), and Bakhtiari and Kashan (2009), who reported that speed tended to increase with advancing age in Quarter Horses, Arabian Horses, and Thoroughbreds, respectively. Performance differences among horses of various ages were attributed, at least in part, to factors such as physiological status (maturity level), training, and prior competition experience (Gómez et al. 2010). A significant effect of sex on barrel racing performance was observed, with females outperforming males. However, the literature lacks consistency regarding this effect across different breeds. In Thoroughbreds, Oki et al. (1994) reported that mares were faster than stallions on turf, but stallions were faster on dirt. Villela et al. (2002) found that male Quarter Horses excelled in time, while females performed better in rank for races up to 503 m. Conversely, Eki̇z et al. (2005) noted superior performance in male Arabian Horses for distances up to 2200 m.

Since individual horses participate in multiple barrel racing competitions throughout their lifetimes, this trait is inherently longitudinal. The present results demonstrate that a cubic RRM provides a better fit for barrel racing performance data compared to a repeatability model. We observed that additive genetic and permanent environmental variances exhibited substantial variation across the age trajectory of the animals, underscoring the need for specific modelling, such as random regression, for these effects. Buxadera and da Mota (2008) employed an RRM to analyse race times of Thoroughbred horses, identifying significant age‐related influences on genetic parameter estimates. Similar findings were reported by Bugislaus et al. (2006) and Gómez et al. (2010), who concluded that the genes influencing speed‐related traits in trotters are age‐dependent. Collectively, these studies, along with our findings, support the use of RRM as appropriate tools for analysing horse performance traits.

The heritability estimates for BRT indicate that this trait can respond effectively to selection at various stages of the animals' lives. The estimates obtained here were comparable to those reported by Villela et al. (2002) for race time (0.17) and by Faria et al. (2023) for earnings, best time, and time class at distances of 301 m and 402 m (0.10–0.37) in Quarter Horses. Our findings aligned with Gómez et al. (2010) for racing speed in Spanish Trotters (0.12–0.34), Bugislaus et al. (2006) for racing speed in German Trotters (0.01–0.18), and Buxadera and da Mota (2008) for race time in Thoroughbreds (0.10–0.22) using RRM. Based on the heritability estimates obtained here, the ages between 36 and 54 months appear most promising for improving barrel racing performance. During this period, heritability estimates were higher, additive genetic variance was more pronounced and BRT showed strong genetic correlations with all other ages, facilitating early selection. It is noteworthy that genetic parameter estimates exhibited wider credibility intervals for extremely young or old ages, likely due to the lower number of performance records available for those age groups.

Genetic correlations for BRT across different ages were generally strong, which, as noted above, supports the potential for effective selection at younger ages. The estimates obtained in this study are consistent with those reported by Bugislaus et al. (2006) for German Trotters, where a genetic correlation of 0.82 was found for racing speed between 2 and 6 years of age. Similarly, Gómez et al. (2010) reported genetic correlations ranging from 0.47 to 0.78 for racing speed in Spanish Trotters between ages 2–4 and 5–8 years across different race distances. Gómez et al. (2010) emphasised that strong and positive genetic correlations between ages represent an advantage for breeders, as selecting the best‐performing horses at younger ages should result in favourable genetic progress at older ages.

The repeatability of BRT was moderate across the entire age trajectory, indicating that horses performing well at younger ages tend to exhibit variability in their performance in subsequent competitions. This finding aligns with the low correlations observed for the permanent environmental effect across divergent ages, suggesting that distinct permanent environmental factors influence performance at different ages. Villela et al. (2002) reported a repeatability estimate of 0.55 for race time in Quarter Horses, which closely matches the value obtained in this study. These findings highlight the complexity of environmental influences on barrel racing performance and the need for age‐specific evaluations.

We identified that the rider effect accounted for an expressive portion of the phenotypic variance. This result was expected, as in barrel racing, the rider's skill in guiding the horse through the course plays a critical role in achieving optimal BRT performance. The importance of riders has been emphasised in various studies on racehorse performance (Buxadera and da Mota 2008; Sharman and Wilson 2023; Velie et al. 2015). However, there is a notable distinction in the dynamics of barrel racing competitions compared to the events and breeds more commonly studied in literature. In Thoroughbreds, Bakhtiari and Kashan (2009) reported that in longer races, riders of leading horses may reduce the horse's speed, contributing additional environmental variation to racing time across different distances and races. Langlois (1996) highlighted that Thoroughbreds race against each other rather than against the clock. Consequently, in European countries, professionals often place little emphasis on speed achievements during animal selection, with such data frequently going unrecorded. In contrast, barrel racing features individual performances where horses and riders compete against the clock, striving to complete the course in the shortest time possible.

No genetic evolution was observed in the BRT of Brazilian Quarter Horses over recent years, which may be explained by the absence of an official breeding program for Quarter Horses and the lack of awareness regarding EBV for barrel racing performance. Therefore, selection practices by Quarter Horse breeders in Brazil have primarily been based on raw performance and awards earned by the animals and their ancestors in competitions. In Thoroughbreds, there has been ongoing debate over whether the performance limits for racehorses have been reached, as evidenced by the lack of improvement in race times over the past few decades (Bailey et al. 2022; Denny 2008; Gaffney and Cunningham 1988). Gardner (2006) reported that, from 1950 to the present, despite significant advances in technology and knowledge enhancing athletic performance, human improvements have led to a 12%–13% reduction in winning times, while Thoroughbred race times have only improved by 4%. In contrast to the observed genetic trend for BRT in the present population, we observed a significant improvement in phenotypic performance for BRT over recent years. This finding aligns with the numerous BRT records broken in the last decade. Therefore, we argue that the advances in BRT performance over recent years have primarily resulted from non‐genetic factors such as management practices, nutrition, training and the increasing competitiveness of events. Thus, the substantial genetic variation identified for BRT in this study is not being fully exploited by breeders to optimise Quarter Horse performance.

## 5 Conclusions

The BRT of Quarter Horses is a heritable trait, exhibiting substantial additive genetic variation, particularly at younger ages, indicating its potential to respond effectively to selection. A random regression model was deemed appropriate for the genetic evaluation of BRT in the studied population. Barrel racing time was influenced by non‐genetic factors such as age, sex and rider. Phenotypically, a notable improvement in BRT performance was observed among Quarter Horses. However, no significant genetic progress was detected over recent years, suggesting that the genetic differences among Quarter Horses for BRT remain underutilised by breeders to enhance competitive outcomes.

## Conflicts of Interest

The authors declare no conflicts of interest.

## Data Availability

The data that support the findings of this study are available from the *Sistema SGP de Gerenciamento de Provas* (https://www.sgpsistema.com).
